# Strikingly low prevalence of pituitary incidentalomas in a teaching hospital in Uruguay

**DOI:** 10.3389/fendo.2023.1254180

**Published:** 2023-09-27

**Authors:** Maria M. Pineyro, Natalia Sosa, Florencia Rivero, Diego Tripodi, Matias Negrotto, Ramiro Lima

**Affiliations:** ^1^Clínica de Endocrinología y Metabolismo, Hospital de Clínicas, Facultad de Medicina, Universidad de la República, Montevideo, Uruguay; ^2^Departamento Clínico de Imagenología, Hospital de Clínicas, Facultad de Medicina, Universidad de la República, Montevideo, Uruguay; ^3^Neurocirugía, Hospital de Clínicas, Facultad de Medicina, Universidad de la República, Montevideo, Uruguay

**Keywords:** pituitary incidentaloma, prevalence, image, headaches, pituitary adenoma, head imaging, sellar mass

## Abstract

**Background:**

Pituitary incidentalomas are an occurrence documented in 10.6% of post-mortem examinations, 4%–20% of computed tomography (CT) scans, and 10%–38% of magnetic resonance imaging (MRI) cases, primarily consisting of microincidentalomas (<1 cm in size). However, the prevalence of pituitary incidentalomas in Uruguay remains unexplored. This study aimed to ascertain the prevalence of pituitary incidentalomas at our hospital.

**Methods:**

In this investigation, we retrospectively identified patients who underwent brain CT and MRI at our hospital over a 1-year span due to conditions other than suspected or known pituitary disorders. The time frame covered was from 1 January to 31 December 2017. Our analysis encompassed all scans, and we conducted interviews with patients discovered to have pituitary incidentalomas. Furthermore, we conducted biochemical assessments in accordance with clinical and imaging traits.

**Results:**

During the study period, a total of 3,894 patients underwent imaging procedures. Of these, 1,146 patients underwent MRI scans, and 2,748 underwent CT scans. The mean age was 53.1 ± 19 years, with a relatively even distribution between genders (50.6% women). The majority of imaging requisitions originated from the emergency department (43%), followed by outpatient clinics (29%), and inpatient wards (28%). Common reasons for imaging requests included trauma (20.4%), headaches (11.3%), and stroke (10.9%). Among these cases, two pituitary incidentalomas were detected, resulting in a prevalence of 5 cases per 10,000 individuals annually (0.051%). Both of these cases were initially identified through CT scans, with subsequent MRI scans performed for further assessment. The final diagnoses were a vascular aneurysm and a sellar meningioma, with the latter patient also exhibiting secondary hypothyroidism. Notably, no instances of pituitary adenomas were encountered.

**Conclusions:**

The prevalence of pituitary incidentalomas within our hospital was notably low. Further research is necessary to more comprehensively investigate the occurrence of pituitary incidentalomas in our country.

## 1 Introduction

A pituitary incidentaloma is an abnormality in the pituitary gland found by brain imaging performed for unrelated indications, in a patient without evident symptoms or signs of pituitary disease (1). They are frequently encountered in clinical practice, owing to the rising usage of neuroimaging technologies. Overall, the prevalence in imaging studies is estimated at an average of 10% in most series, with a range between 1% and 30% in different studies (2, 3). The reported occurrence of pituitary incidentalomas spans from 10.7% in 18,902 autopsies to 12.6% within 485 Iranian post-mortem investigations (4, 5). In addition, in three series of adult patients who underwent computed tomography (CT) scans, microinicidentalomas (defined as focal hypodensity >3 mm) were reported in 4%–20% of cases (6–8). Moreover, incidental microincidentalomas (<1 cm) were reported in 10%–38% of adult patients who underwent magnetic resonance imaging (MRI) (9, 10). Macroincidentalomas have been found in 0.16%–0.3% of MRIs performed in a healthy population (11, 12), as well as in 0.2% of 3,550 individuals who underwent CT in an observational study (13). Pituitary incidentalomas may present with autonomous hormonal activity in pituitary adenomas or impair normal gland function. Ninety percent of lesions presenting as incidentalomas are pituitary adenomas and Rathke cleft cysts (14, 15). In addition, the reported frequency of craniopharyngiomas is approximately of 5% (16). In a sequence of surgical procedures performed on sellar masses, most (91%) were pituitary adenomas (17). The rest were non-adenoma pathologies, the most common Rathke’s cysts, and craniopharyngiomas. Regarding the different types of incidental pituitary adenomas, one study reported 77% of non-functioning adenomas, 18% of prolactinomas, and 3% GH-secreting adenomas (15). The prevalence of pituitary incidentalomas in Uruguay is not known. Our objective was to assess the prevalence of these incidentalomas within our hospital.

## 2 Materials and methods

We conducted a retrospective analysis of all patients who underwent brain CT and MRI scans at our hospital over the course of 1 year, excluding cases related to recognized or suspected pituitary disorders. The definition of pituitary incidentaloma was considered according to the Endocrine Society guidelines as any pituitary lesion in imaging studies indicated for unrelated reason (1). The time frame considered spanned from 1 January to 31 December 2017. Excluded from the study were patients with a prior diagnosis of pituitary pathology, pregnant women, and those whose imaging was requested specifically for visual or pituitary symptoms. For data collection, both the imaging report and the images were analyzed by two of the authors, which was then further evaluated by a third-year imaging fellow or an assistant professor of imaging. This review was not independent or blinded. We collected demographic and clinical data from clinical records. We examined all scans and conducted an anamnesis for patients who exhibited pituitary incidentalomas, and biochemical evaluation was done according to clinical and image characteristics. The analyzed images were obtained using a 64-row Siemens Somatom Sensation Tomograph and a Siemens Magnetom Avanto 1.5-tesla Resonator. They were made with 2-mm cuts in a standard manner. Those patients whose images were qualified as a pituitary adenoma or whose anatomical relationship compromised or contacted the pituitary gland or the pituitary stalk in some way were referred to the Endocrinology and Metabolism Department for evaluation and follow-up. Hormonal dysfunction was determined by laboratory analysis with basal IGF-1, FSH, LH, total testosterone/estradiol, TSH, free T4, morning cortisol, and prolactin levels. Written informed consent was obtained. Data are presented as frequencies, mean, standard deviation, and range. The prevalence was calculated as the number of cases of pituitary incidentaloma divided by the total number of images during the study period. Chi-square test was used to evaluate the association of categorical variables and the Student’s *t*-test was performed to assess differences between means for independent samples. A *p*-value of less than 0.05 was considered statistically significant. The study was approved by the Ethics Committee of the Hospital de Clínicas. The study was carried out according to the standards of the Medical Ethics Committee of the Faculty of Medicine, and principles of the Helsinki Declaration.

## 3 Results

We analyzed a total of 3,894 imaging studies, most of which were CT (71%). The mean age of patients was 53.1 ± 19 years (range 12–99). Patients who underwent CT were significantly older compared to those who underwent MRI (53.7 ± 0.4 vs. 51.5 ± 0.5 years, respectively, *p* = 0.001). There was a similar distribution between genders: 1,923 patients (49.4%) were men and 1,971 (50.6%) were women.

**Figure 1:**
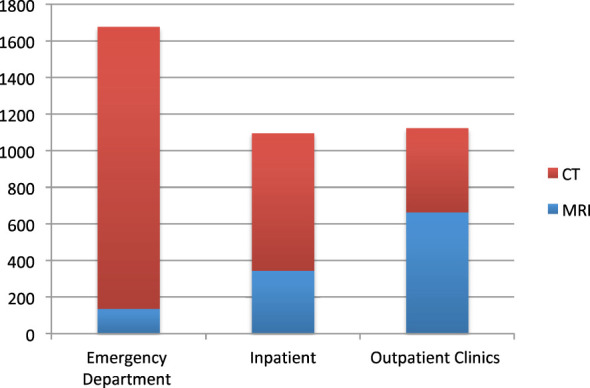
Type of image according to requesting department. CT, computed tomography; MRI, magnetic resonance imaging.

Most of the images were requested by the emergency department (43%), followed by the outpatient clinics (29%) and finally the wards (28%). The CT was more frequently requested in the emergency department, while the MRI was requested in the outpatient clinics (**Figure 1**).

If we take into account all the images, the most frequent reason for requesting the imaging was head trauma and polytrauma (20.4%), followed by headache (11.3%). Headache was the most frequent reason for requesting MRI, and head trauma and polytrauma were that for CT (**Table 1**).

**Table 1:** Indications for head/brain imaging.

Reason	MRI (*n*)	CT (*n*)	Total
Head trauma and polytrauma	12	784	796(20.4%)
Headache	106	334	440 (11.3%)
Stroke	95	328	423 (10.9%)
Seizure	83	117	200 (5.1%)
Cognitive impairment	28	64	92 (2.4%)
Other (metastases, altered states of consciousness, and central nervous system infections)	609	957	1,566 (40.2%)
Missing or unknown	213	164	377 (9.7%)
Total	1,146	2,748	3,894 (100%)

Two cases of pituitary incidentalomas were found by means of CT, which corresponds to a prevalence of 5 cases per 10,000 studied per year (0.051%). No pituitary incidentalomas were found on MRI. We describe the two cases found.

### 3.1 Patient 1

**Figure 2:**
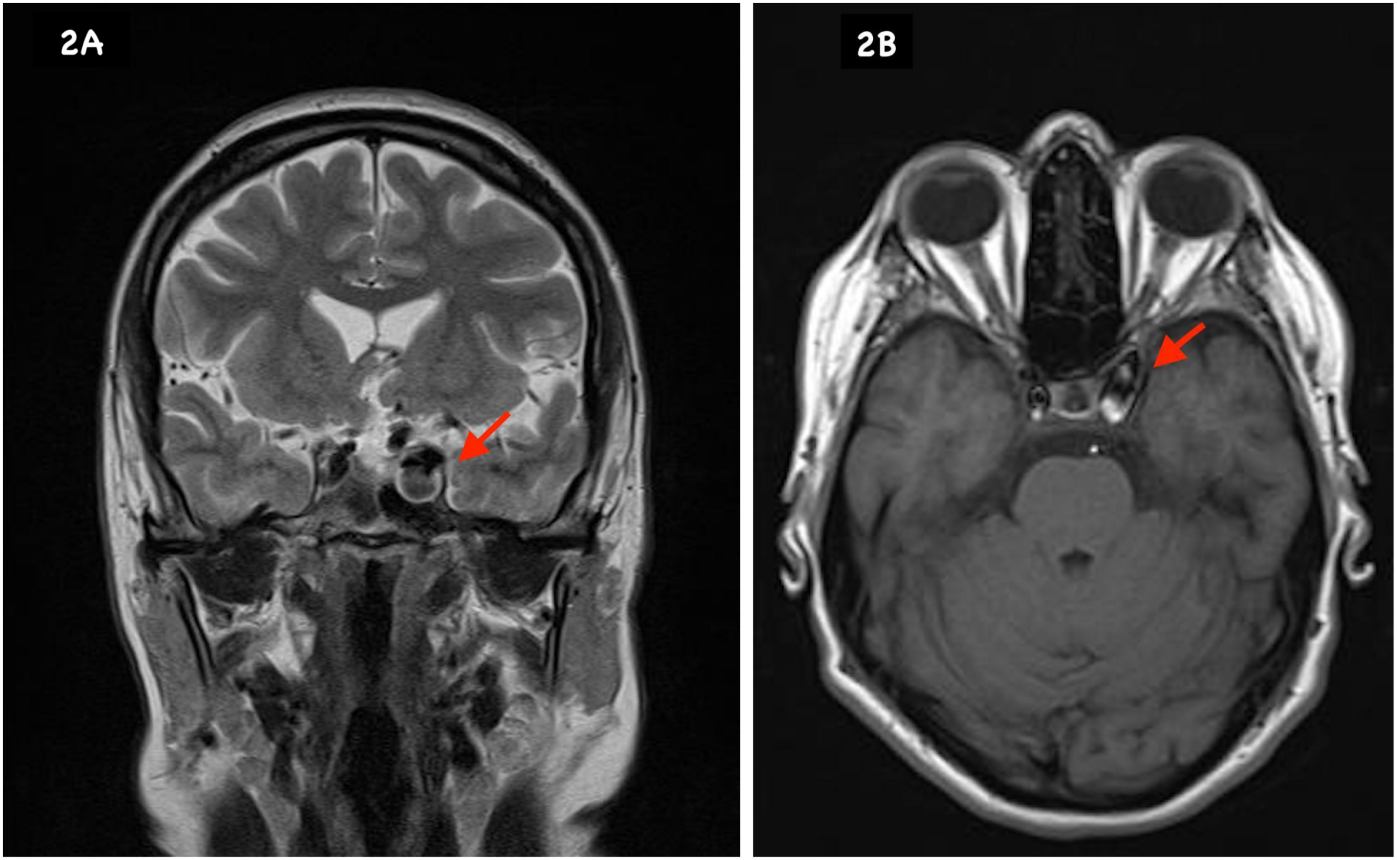
MRI T2 **(A)** and T1 **(B)** show a carotid-ophthalmic aneurysm.

A pituitary incidentaloma was found by CT scan in a 46-year-old female patient who was worked up for headaches. Evaluation was completed by magnetic resonance angiography, and a diagnosis of a carotid-ophthalmic aneurysm was made (**Figure 2**). Given that the lesion did not exhibit anatomical proximity to the pituitary gland or the pituitary stalk, hormone workup was not performed.

### 3.2 Patient 2

**Figure 3:**
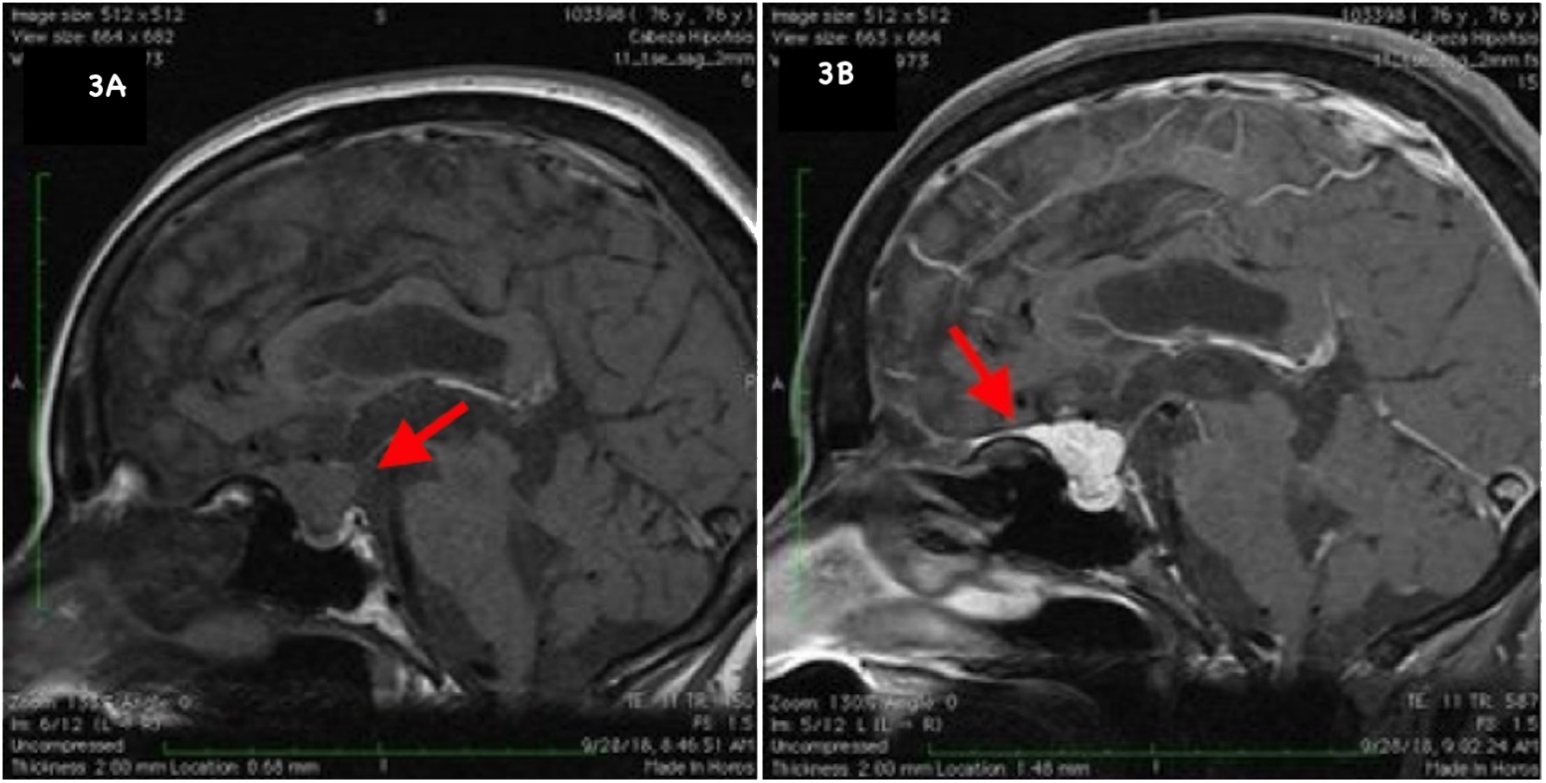
Sellar MRI. Sagittal **(A)**: T1 pre-contrast shows an isointense suprasellar expansive process with intrasellar extension. **(B)**: Hyperintense lesion after gadolinium administration. Dural tail sign, characteristic of sellar meningiomas.

A pituitary incidentaloma was found in a CT scan in a 76-year-old female patient being evaluated for tinnitus and hearing loss. MRI showed a 21 × 17 × 15mm sellar meningioma, contacting the optic chiasm and the pituitary stalk (**Figure 3**).

The patient had no symptoms. Hormonal evaluation revealed secondary hypothyroidism with TSH values of 4.38 pg/mL (normal range: 2.0–4.4 pg/mL) and FT4 values of 0.83 ng/dL (reference: 0.93–1.7 ng/dL), with intact corticotropic axis. Prolactin levels were within normal limits.

## 4 Discussion

In our study conducted in Uruguay, we identified an annual prevalence of pituitary incidentalomas at a rate of 5 cases per 10,000 individuals (0.051%), which corresponds to 2 cases within a dataset of 3,894 images (1,146 MRI scans and 2,748 CT scans). We noticed a surprisingly lower prevalence of pituitary incidentalomas in contrast to what has been documented in the existing literature. Furthermore, there were no occurrences of pituitary incidentalomas detected by MRI scans. Additionally, the absence of pituitary adenomas was notable. The underlying causes for these observations remain uncertain.

The most common indications in this study for head imaging were head trauma and headaches. For MRI, headache was the most common indication for initial imaging, and for CT, the most common indications were head trauma and polytrauma. According to the findings of Imran et al., the predominant cause for imaging was headache in 328 pituitary incidentalomas found in MRI/CT scan in two referral centers of Canada (18). In addition, it was also the indication for imaging of 506 pituitary incidentalomas found in MRI/CT scans in Japan (19). Furthermore, headaches were the primary reason for undergoing imaging, leading to the detection of 459 non-functioning pituitary microadenomas across 23 endocrine departments in the United Kingdom (20). Headache has been a symptom associated with small and large non-functioning and functioning pituitary tumors. It has been reported in up to 70% of patients. They are believed to be due to stretching of the dura and activation of afferent pain fibers, although tumor size has not been shown to be associated with headaches (21). Other proposed mechanisms include hormone hyper or hyposecretion and increased intrasellar pressure (22). There is a controversy as to whether headaches are associated with pituitary incidentalomas.

The prevalence of pituitary incidentalomas detected in CT scans of patients who have experienced trauma varies, ranging from no detections to approximately 0.5% of cases. For example, in an evaluation of 3,000 brain CT scans indicated for head trauma, they found eight tumors (three meningiomas, two craniopharyngiomas, one oligodendroglioma, one low-grade astrocytoma, and one medulloblastoma) (23). They did not find pituitary adenomas. In this study, CT scans were low quality for pituitary microadenoma detection (CT 10 mm thick slice), and patients were young with a mean age of 32 ± 17 years. In the sellar region, they found only 2 craniopharingeomas. Also, in 600 CT scans done in patients with head injuries, 12 brain tumors were found (2%), mostly meningiomas. In this study, three pituitary macroadenomas were reported (0.5%) (24). In addition, in 991 patients who had CT scans for trauma, no pituitary incidentaloma was found (25). In 591 CT scans done in patients with head trauma, 0.7% of brain tumors were reported, mostly meningiomas. Only one pituitary adenoma presenting as pituitary apoplexy was found (26). In addition, in 321 total-body CT scan done in trauma patients, no pituitary lesions were found (27).

In our investigation, the majority of CT scans were carried out within the emergency department. Four meningiomas were found in 405 head CT scans conducted in the emergency department (28).

On average, imaging reveals the presence of pituitary incidentalomas in approximately 10% of cases. A meta-analysis of five studies reported a prevalence of pituitary incidentalomas of 22% (range 10%–38%) (29). In recent population-based studies, the prevalence was 15–21/100,000 in the general population, and they represented 16%–36% of pituitary adenomas (30–33).

Some studies have reported lower frequencies of pituitary incidentalomas. Kuo et al. reported incidental sellar findings in only 45 (1.2%) of 3,840 head CT/MRI done at Cedar-Sinai Medical Center. Of these 45 findings, most were empty sella, with only one sellar meningioma, one Rathke cyst, and two pituitary adenomas (4/3,840, 0.1%). In addition, pituitary adenomas were found with MRI imaging (34). In a longitudinal, population-based study of cardiovascular and cerebrovascular disease, Yue et al. documented a prevalence of 0.16% for pituitary adenomas among 3,672 MRIs performed on patients aged 65 years and older (11). Nammour et al. reported a prevalence of 0.2% (95% confidence interval 0.05%–0.35%) pituitary macroadenomas in 3,550 CT scans at the Cleveland Department of Veterans Affairs Medical Center (13). Two pituitary incidentalomas were found in 700 (0.28%) MRIs performed among older individuals residing within a community setting (mean age of 72.5± 1.5 years) of Edinburgh (35). In 1,006 MRIs done in healthy adult volunteers, three pituitary incidentalomas (0.3%) were observed (36). Moreover, nine pituitary incidentalomas and four pituitary cysts (0.52%) were reported in 2,500 whole-body MRI done to residents of Germany (37). Furthermore, among 318 cone beam CT scans conducted for dental implant purposes, two cases of pituitary incidentalomas were identified, accounting for a prevalence of 0.63% (38). Vernooij et al. found 1.6% pituitary tumors in 2,000 brain MRIs performed to persons from the population-based Rotterdam Study (12).

Within this study, the majority consisted of CT scan images, although this modality is not the preferred choice for comprehensive evaluation of pituitary lesions. In addition, CT scan images were indicated for head trauma and polytrauma, in the emergency room. To our knowledge, only few pituitary incidentalomas were reported in these settings.

We did not find any pituitary incidentaloma on MRI scans. In our study, the scans were not specifically targeted towards the pituitary fossa, which could potentially result in the oversight of small lesions. However, most of the studies reporting pituitary incidentalomas are with brain MRIs, not focused sellar MRI (11, 39). For example, Esteves et al. documented a 5.8% occurrence rate of pituitary incidentalomas among 1,232 patients who underwent head MRI/CT scans, rather than pituitary MRI scans. Notably, the majority of these cases were attributed to pituitary adenomas, of which nearly 40% were microadenomas.

In our research, scan slices with a 2-mm thickness were acquired, following imaging methods comparable to those employed in other investigations. The majority of reports typically encompass more extensive study periods, spanning 3 to 5 years. It is noteworthy that our study took place within a teaching hospital environment, where initial scan assessments are conducted by residents and fellows. Subsequently, these evaluations are subjected to reevaluation by neuroradiologists. This situation might explain the observed prevalence, given that the sensitivity of scan interpretations could potentially be reduced when carried out by professionals lacking medical qualifications. Nevertheless, it is plausible that the frequency of pituitary hypointensity areas could decrease with an increasing number of reviewers involved.

Hall et al. reported 10 microadenomas in 100 MRIs done in healthy volunteers interpreted by two blinded reviewers, which decreased to 2 adenomas when revised by three blinded reviewers (40). This was a retrospective study. The images had already been interpreted by an assistant professor or associate professor for MRIs, and by a third-year imaging fellow or an assistant professor of imaging for CT scans. These interpretations were reported in the patient chart as part of routine care. In our study, two of the authors reviewed both the report and the image, which was then further evaluated by a third-year imaging fellow or an assistant professor of imaging. However, it was not an independent or blinded review.

However, various factors may affect interpretation of scans such as experience levels, measurement limits, image quality, and omitted clinical information (41). In addition, in some series reporting non-functioning pituitary microincidentalomas, approximately 15%–25% have one or more pituitary hormone deficits that can be attributed to the lesion (42–45), as well as approximately 10% have hyperprolactinemia (42, 43). If those pituitary incidentalomas were to be excluded by a previous evaluation, frequency can be lower. Moreover, this reduced occurrence might be linked to variations in the demographic attributes of the population.

There are some limitations in our study. Although the sample size is large, it is a retrospective single-center study. In addition, we did not collect data regarding the number of contrast-enhanced images. It should be noted that microadenomas imaged without contrast might pose challenges in their identification. Notably, a majority of studies reporting low frequencies of pituitary incidentalomas were conducted without the utilization of contrast material (11–13, 35, 36, 38). While we did not collect data on the use of contrast material in imaging, we hold the firm belief that our findings regarding the prevalence of pituitary incidentalomas in Uruguay remain highly significant. Our results may not be generalizable to other institutions.

## 5 Conclusion

A notably low frequency of pituitary incidentalomas was uncovered within our hospital. Additional studies are needed to delve deeper into the prevalence of these incidentalomas across our nation.

## Data availability statement

The raw data supporting the conclusions of this article will be made available by the authors, without undue reservation.

## Ethics statement

The studies involving humans were approved by Ethics Committee of the Hospital de Clínicas,. The studies were conducted in accordance with the local legislation and institutional requirements. The participants provided their written informed consent to participate in this study.

## Author contributions

MP: Conceptualization, Formal Analysis, Supervision, Writing – original draft, Writing – review & editing. NS: Data curation, Formal Analysis, Investigation, Methodology, Writing – review & editing. FR: Data curation, Formal Analysis, Investigation, Methodology, Writing – review & editing. DT: Data curation, Formal Analysis, Investigation, Methodology, Writing – review & editing. MN: Data curation, Formal Analysis, Investigation, Methodology, Writing – review & editing. RL: Formal Analysis, Supervision, Writing – review & editing.
